# A comprehensive meta-analysis of circulation miRNAs in glioma as potential diagnostic biomarker

**DOI:** 10.1371/journal.pone.0189452

**Published:** 2018-02-14

**Authors:** Chenkai Ma, Hong P. T. Nguyen, Rodney B. Luwor, Stanley S. Stylli, Andrew Gogos, Lucia Paradiso, Andrew H. Kaye, Andrew P. Morokoff

**Affiliations:** 1 Department of Surgery, The University of Melbourne, The Royal Melbourne Hospital, Parkville, Victoria, Australia; 2 Department of Neurosurgery, The Royal Melbourne Hospital, Parkville, Victoria, Australia; Thomas Jefferson University, UNITED STATES

## Abstract

Glioma is the most common malignant intracranial tumour. Recently, several publications have suggested that miRNAs can be used as potential diagnostic biomarkers of glioma. Here we performed a meta-analysis to identify the diagnostic accuracy of differentially expressed circulating miRNAs in gliomas. Using PubMed, Medline and Cochrane databases, we searched for studies which evaluated a single or panel of miRNAs from circulating blood as potential biomarkers of glioma. Sixteen publications involving 23 studies of miRNAs from serum or plasma met our criteria and were included in this meta-analysis. The pooled diagnostic parameters were calculated by random effect models and overall diagnostic performance of altered miRNAs was illustrated by the summary receiver operator characteristic (SROC) curves. The pooled sensitivity, specificity, positive likelihood ratio (PLR) and negative likelihood ratio (NLR) from each study were calculated. The pooled PLR, NLR and Diagnostic Odds Ratio were 6.39 (95% CI, 4.61–8.87), 0.15 (95% CI, 0.11–0.21) and 41.91 (95% CI, 23.15–75.88), respectively. The pooled sensitivity, specificity and area under the curve (AUC) were 0.87 (95% CI, 0.82–0.91), 0.86 (95% CI, 0.82–0.90) and 0.93 (95% CI, 0.91–0.95), respectively. This meta-analysis demonstrated that circulating miRNAs are capable of distinguishing glioma from healthy controls. Circulating miRNAs are promising diagnostic biomarkers for glioma and can potentially be used as a non-invasive early detection.

## Introduction

Glioma, the most prevalent malignant cancer in the central nervous system, has a content of latent progression before histopathological diagnosis [1]. The incidence of glioma in adults varies from 1.32 to 5.73 per 100, 000 person, depending on geographic location [2]. Glioma is categorized according to the World Health Organization (WHO) grading system, grade I and II are classified as benign and low grade glioma while grade III and IV are high grade glioma. The current protocolized treatment for high grade glioma is surgery followed by concurrent radiotherapy and temozolomide chemotherapy [3]; despite this, glioma remains a highly aggressive cancer with extremely poor prognosis, resulting in median survival of less than 15 months. Glioma recurrence is almost always seen and is usually accompanied with higher malignancy and infiltrative growth, causing rapid progression of disease and larger community-family burden [4] with 5-year survival rate remaining less than 3% [5].

Currently, contrast-enhanced magnetic response imaging (MRI) is a useful guide for pre-surgical diagnosis and post-surgical monitoring of glioma. It cannot detect micro lesions nor provide all of the biological information regarding the tumour [6, 7]. Furthermore, it is expensive and inconvenient for many patients. Histology is the basis of glioma diagnosis and prognosis, but requires invasive neurosurgery to obtain tumour tissue. In light of this, there is urgency to discover an improved minimally invasive and rapid approach for diagnosis and monitoring of glioma.

Biomarkers from bio-fluid have been widely studied in clinical diagnosis and disease monitoring in cancer and some are in clinical use. MicroRNAs (miRNAs), a family of small non-coding RNAs (19–22 nucleotides), have been shown to take part in multiple biological activities such as immunology regulation, embryo development and cancer progression through post transcriptional modifications. Its mechanism of action is to bind the complimentary 3’ UTR of target mRNAs and degrading the mRNAs [8, 9]. Deregulated miRNAs can be detected in the surgical specimens and the bio-fluid like serum, plasma, urine and cerebrospinal fluid (CSF) from patients, suggesting altered miRNAs have the potential to become a non-invasive biomarker for cancer detection [10, 11]. Recently, several studies have shown that a single, or panel of, circulating miRNAs can be used as diagnostic tools for several cancers including bladder cancer, hepatocellular carcinoma, lung cancer, cervical cancer, ovarian cancer, gastric cancer and colorectal cancer with high sensitivity and specificity [12–18]. The expression of circulating miRNA has also been correlated with patient outcome [19]. The diagnostic and prognostic utility of miRNAs in bio-fluid for glioma, however, remains ill-defined. We performed a meta-analysis to assess the published clinical studies about circulating miRNAs in glioma patients in order to better evaluate this biomarker.

## Methods

### Search strategy

We performed a comprehensive search for original papers demonstrating diagnostic power of circulating miRNAs in patients with glioma. Databases including Pubmed, Embase, the Cochrane Library Database and Google Scholar were used to obtain all relevant studies up to February 2017. Key words including “glioma” or “glioblastoma”, “microRNA” or “miRNA” or “miR” and “plasma” or “serum” or “circulating” or “blood”, which worked as Medical Subject Headings (MeSH) and free-style words in searching retrieval. No publication date was restricted in our search. All publications containing the relevant keywords were included in this meta-analysis.

### Inclusion and exclusion criteria

Selection criteria have been set up for literature selection before performing meta-analysis. The inclusion criteria: (i) studies aim to identify the diagnostic capacity of miRNAs for glioma, (ii) all patients must be diagnosed as glioma by using the gold standard test (namely by histopathology examinations), (iii) sample sizes, sensitivity, specificity or sufficient data were reported in the original studies to analyze the diagnostic ability and (iv) studies that used plasma or serum as the source of study materials. Exclusion criteria: duplicate publications, insufficient data provided, reviews, letters, editorials and commentaries and studies on miRNAs in cerebrospinal fluid (CSF).

The studies considered satisfactory and included in this meta-analysis all have glioma indication as patient cohort and compared with non-glioma controls. Characteristics of included studies were collected as following: first author’s name, year of publication, numbers of cases and controls, types of glioma, methods used to detect miRNAs, miRNA names, sources of samples, the sensitivity and specificity, the test for true positive (TP), false positive (FP), false negative (FN) and true negative (TN) and any other additional information required for quality evaluation. Values obtained for these parameters were additionally calculated using Revman 5 software (Review Manager, version 5.3. Copenhagen: The Nordic Cochrane Centre, The Cochrane Collaboration, 2014). The methodology quality (risk of bias and applicability of primary diagnostic accuracy) of each trial was evaluated by the revised Quality Assessment of Diagnostic Accuracy Studies (QUADAS-2) tool [20].

### Diagnostic capacity analysis

All statistical analysis was conducted using Stata software version 13 (StataCorp LCC, Texas USA). The number of participants with TP, FP, FN and TN were extracted in order to calculate the pooled sensitivity, specificity, positive likelihood ratio (PLR), negative likelihood ratio (NLR), diagnostic odds ratio (DOR) and corresponding 95% confidence intervals (CIs). The summary receiver operator characteristic (SROC) curve and area under the SROC curve (AUC) were evaluated to test the pooled diagnostic performance of circulating miRNAs reported from original studies. Deek’s funnel plot was conducted to assess the publication bias in our meta-analysis. A *p* value less than 0.05 was considered statistically significant.

## Results

### Studies characteristics

**Fig 1:**
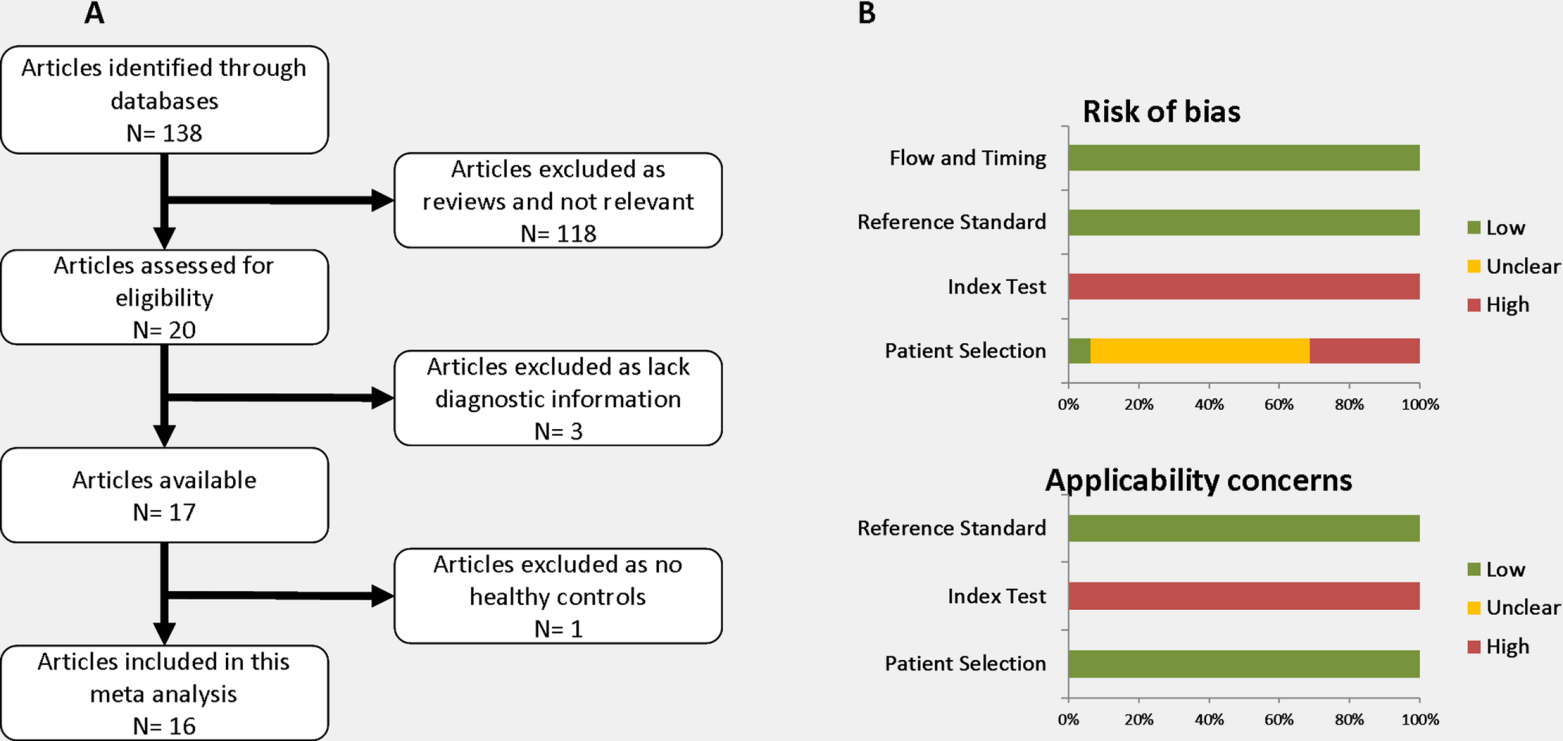
Flow chart of studies selection and quality assessment of studies. **A)** A total of 16 published articles were included in this meta-analysis after filtering through the inclusion criteria. **B)** QUADAS-2 assessment. Bar chart showing the summary of risk of bias and applicability concerns, expressed as percentage. Each study occupied the bar equally (1/16, 6.25%). Red: high risk; Yellow: unclear risk; Green: low risk.

Using our retrieval strategy, a total of 138 publications were likely identified as relevant studies about miRNAs as potential biomarkers of glioma (**Fig 1A**). By reviewing the abstracts, 118 were excluded as they are reviews and/or studies unrelated to glioma diagnostic analysis topic. Of the remaining 20 publications, three studies were also removed as they failed to provide diagnostic information. One other article was also excluded as it studied diagnostic accuracy of circulating miRNA between glioblastoma and low grade glioma rather than miRNA biomarker in tumour and normal control. Consequently, 16 publications met our inclusion criteria and were included in this meta-analysis for [21–33] [29, 34, 35] **(Fig 1A)**.

The 16 publications (ranging from year 2011 to 2017) included in this meta-analysis reported 23 cohorts, totaling 2325 participants. Seven publications reported a panel of several miRNAs as a diagnostic biomarker, while nine studies discussed a single miRNA candidate **(Table 1)**. D’Urso et al, Manterola et al, Zhang et al and Zhi et al reported two study cohorts in each of their articles [22, 32–34]. Articles by Wang et al and Huang et al included a subset of three studies [26, 35]. The remaining articles reported one study each. In all studies, the level of miRNA expression in plasma (n = 5), serum (n = 10) or blood (n = 1) was detected by quantitative real-time polymerase chain reaction (qRT-PCR).

**Table 1:** Characteristics of included circulating miRNA studies in this meta analysis.

Study	Year	Case size	Control size	Cohort Source	Cancer type	Methodology	miRNA Signatures (up- or down-regulated in glioma)	Source	Normalization
D’Urso et al	2015	30	30	Training Set and Validation Set	Glioma (grade I to IV)	qRT-PCR	miR-15b (up)	Plasma	miR-24
Huang et al	2017	100	50	Training Set	Glioma (grade I to IV)	qRT-PCR	miR-376a, miR-376b, miR-376c (down)	Serum	RNU6
Lai et al	2015	126	40	Validation Set	Glioma (grade I to IV)	qRT-PCR	miR-210 (up)	Serum	miR-16-1
Manterola et al	2014	75	55	Training Set and Validation Set	Glioblastoma (grade IV)	qRT-PCR	RNU6-1, miR- 320, miR-574-3p (up)	Serum	RNU48
Regazzo et al	2016	15	10	Training Set	Glioblastoma (grade IV)	qRT-PCR	miR-497, miR-125b (down)	Serum	UniSP2
Roth et al	2011	20	20	Validation Set	Glioblastoma (grade IV)	qRT-PCR	miR-128 (up), miR-342-3p(down)	Blood	RNU48
Shao et al	2015	70	70	Training Set	Glioma (grade I to IV)	qRT-PCR	miR-454-3p (up)	Plasma	cel-miR-39
Sun et al	2015	151	53	Training Set	Glioma (grade I to IV)	qRT-PCR	miR-128 (down)	Serum	cel-miR-39
Wang et al	2012	10	10	Training Set	Glioblastoma (grade IV)	qRT-PCR	miR-21 (up) miR-128, miR-343-3p (down)	Plasma	mmu-miR-295
Wei et al	2014	33	33	Training Set	Glioma (grade I to IV)	qRT-PCR	miR-125b (down)	Serum	miR-24
Wu et al	2014	83	69	Training Set	Glioma (grade I to IV)	qRT-PCR	miR-29 (down)	Serum	miR-24
Xiao et al	2016	112	54	Training Set	Glioma (grade I to IV)	qRT-PCR	miR-182 (up)	Plasma	RNU6B
Yang et al	2012	133	80	Training Set and Validation Set	Astrocytoma (grade II to IV)	qRT-PCR	miR-15b*, miR-23a, miR-133a, miR-150*, miR-197, miR-497, miR-548b-5p (down)	Serum	Serum Volume
Yue et al	2016	64	45	Training Set	Glioma (grade I to IV)	qRT-PCR	miR-205 (down)	Serum	miR-16
Zhang et al	2015	50	51	Training Set	Glioma (grade I to IV)	qRT-PCR	miR-221/222 (up)	Plasma	miR-16
Zhi et al	2015	140	160	Training Set and Validation Set	Astrocytoma (grade II to IV)	qRT-PCR	miR-15b-5p, miR-16-5p, miR-19a-3p, miR-19b-3p, miR-20a-5p, miR-106a-5p, miR-130-3p, miR-181b-5p, miR-208a-3p (up)	Serum	Serum Volume

Of the 16 studies analyzed (Table 1), 5 publications [22, 23, 30, 33, 34] tested global miRNA expression using a panel of miRNA available commercially including those using the Taqman Low Density Array (TLDA) based on different versions of miRNA databases from mirBase [22, 33]. Nine publications only focused on a targeted miRNA that has previously been assessed as potential biomarker or diagnostic marker in glioma or other cancer types (**Table 1**). Some studies used healthy volunteers as their controls, while one study included patients with other neurological conditions as controls [34]. All studies collected biofluid at the time of glioma diagnosis or prior to operation, however some studies also included blood collected post-surgery.

### Potential miRNA biomarkers

There were several miRNAs identified more than once among the studies in this meta-analysis (**Table 1**). The most common differentially expressed miRNAs between glioma and healthy controls were miR-15b [30, 34] and miR-125b [27, 36] (**Table 1**). The direction of differential expression was contradictory between some reports; notably, miR-128 was reported as significantly down-regulated in two studies [25, 26], but up-regulated in a third publication [23].

The QUADAS-2 tool was used to review the risk of bias and applicability for each included article, where all included records displayed a moderate quality **(Fig 1B)**. The highest risk after assessment was from the index test because there was a large variance between the technical methods, or the thresholds that the original studies used. Since it is not clinically warranted to perform ‘screening’ miRNA testing without surgical biopsy, blinding of miRNA results to histopathological diagnosis may not have been reliable, which eventually raises the risks of testing assessment.

The reference genes chosen to normalize in the studies also contributed to the risks. Noticeably, all studies were assessed as high risk in index test sections as they used different endogenous genes or miRNAs as control for normalization **(Table 1)**. The difference in the report of miRNA-128 expression between studies may be caused by the use of different reference genes (i.e. RNU6 in Roth’s study and spiked-in miRNA in Wang and Sun’s studies). Overall, RNU6 (including RNU6B) was the most common reference gene used for normalization, followed by miR-16. Interestingly, RNU6-1 is reported to be an up-regulated biomarker in another study [22] that compared glioma to control serum. This variability in normalization due to lack of well-established guidelines for assessing circulating miRNA, contributed to an increase in risk of bias and heterogeneity of studies.

### Diagnostic accuracy

**Fig 2:**
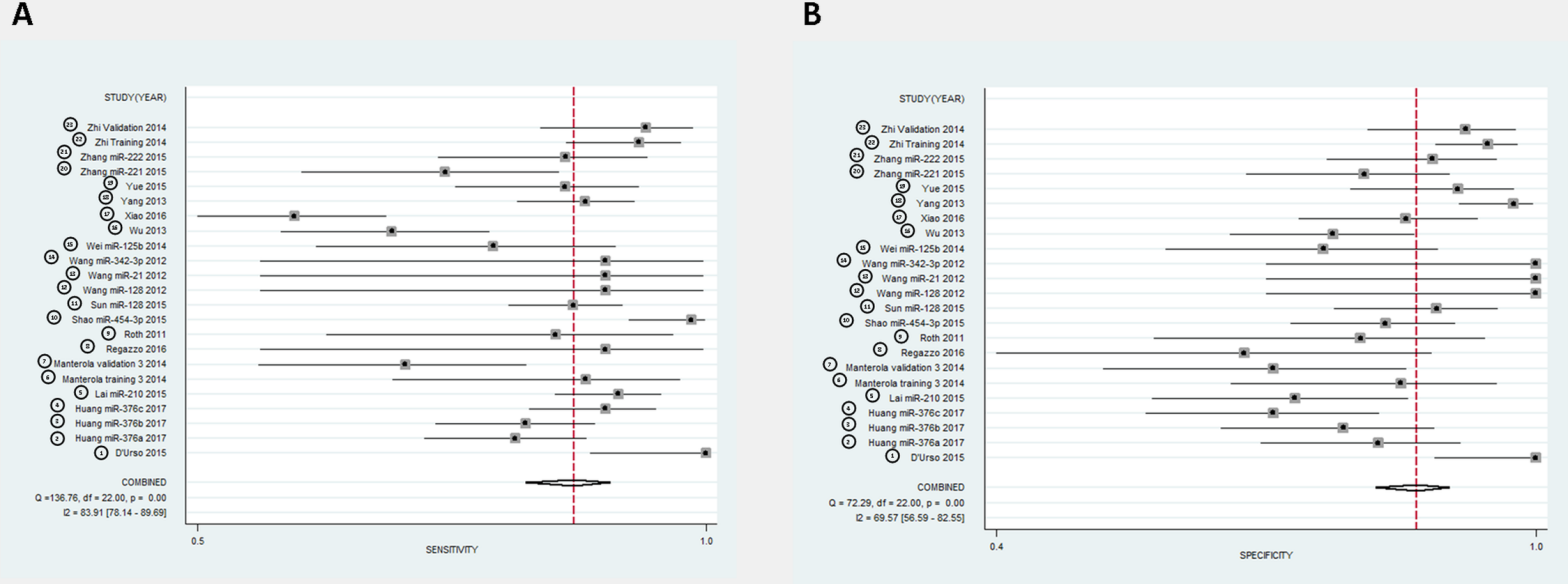
Forest plots of all 16 studies included in this meta-analysis. The pooled **A)** sensitivity and **B)** specificity is 0.87 and 0.86, respectively of miRNAs in diagnosis of glioma.

**Fig 3:**
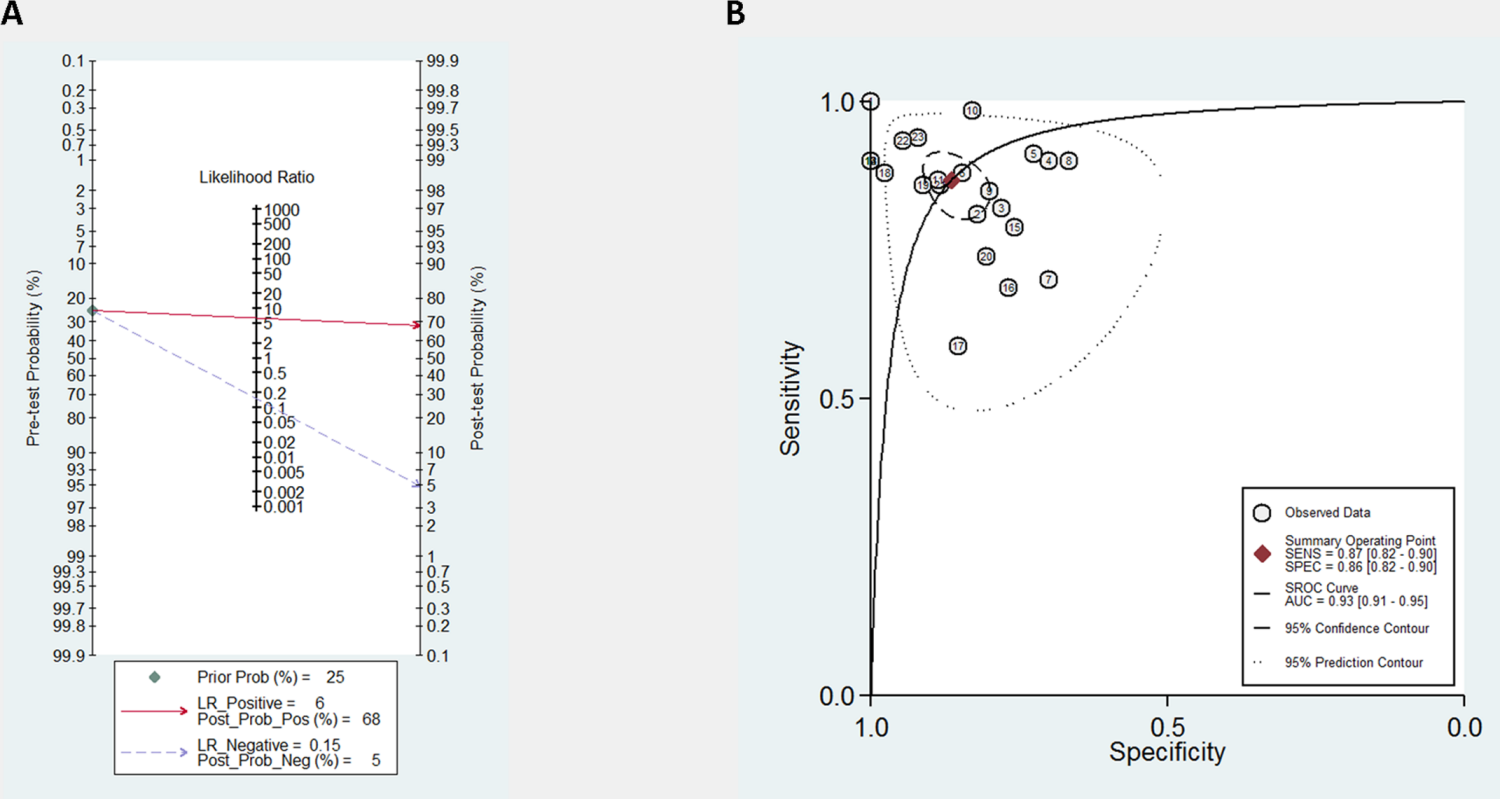
Diagram of SROC curves describing the diagnostic performance of miRNAs. **A)** The PLR and NLR is 6.39 and 0.15 respectively, showing the pre-test probability set as 25%, the positive and negative post-test probability of 68% and 5%, respectively. **B)** The AUC is 0.93 (95%CI, 0.91–0.95). Each number within a circle represents the order of study identifier in Fig 3.

Diagnostic accuracy, sensitivity and specificity of each dysregulated single miRNA or miRNAs panel are summarized in **Table 2**. Forest plots of pooled data (23 studies from 16 articles) demonstrated a large heterogeneity of included studies (**Fig 2**). Thus, the mixed effect model was applied to yield the sensitivity (0.87 (95% CI, 0.82–0.90) **(Fig 2A).** and specificity 0.86 (95% CI, 0.82–0.90) **(Fig 2B)** of this meta-analysis. The Diagnostic Odds Ratio (DOR) was identified as 41.91 (95% CI, 23.15–75.88) and the pooled PLR and NLR were 6.39 (95% CI, 4.61–8.87), 0.15 (95% CI, 0.11–0.21), respectively **(Fig 3A)**. In addition to diagnostic accuracy, the summary receiver operator characteristic (SROC) curve was plotted and the Area Under the Curve (AUC) was 0.93 (95% CI, 0.91–0.95) **(Fig 3B)**, suggesting that circulating miRNAs may be valuable as diagnostic tool. Subtype analysis was also performed according to the type of sample used (serum and plasma) (**S1 Fig)**.

**Table 2:** Diagnostic characteristics of each single miRNA and miRNA panel.

miRNA(s)	Expression	Sensitivity	Specificity	AUC	Study
Single miRNA					
miR-15b	Upregulated	98%	98%	0.98	D’Urso et al, 2015
miR-376a	Downregulated	81%	82%	0.872	Huang et al, 2017
miR-376b	Downregulated	82%	78%	0.890	Huang et al, 2017
miR-376c	Downregulated	90%	70%	0.837	Huang et al, 2017
miR-182	Upregulated	58.5%	85.2%	0.778	Xiao et al, 2016
miR-128	Downregulated	86.75%	88.68%	0.9095	Sun et al, 2015
		90%	100%	1	Wang et al, 2012
miR-29	Downregulated	68.5%	77.3%	0.74	Wu et al, 2014
miR-125b	Downregulated	78.79%	75.76%	0.839	Wei et al, 2014
miR-210	Upregulated	91.27%	72.50%	0.927	Lai et al, 2015
miR-454-3p	Upregulated	99.05%	82.86%	0.9063	Shao et al, 2015
miR-21	Upregulated	90%	100%	0.93	Wang et al, 2012
miR-342-3p	Downregulated	90%	100%	1	Wang et al, 2012
miR-205	Downregulated	86.3%	92.2%	0.935	Yue et al, 2015
miR-221	Upregulated	73.5%	80%	0.83	Zhang et al, 2015
miR-222	Upregulated	85.7%	87.5%	0.88	Zhang et al, 2015
MiRNA panel					
miR-497, miR-125b	Downregulated	88.9%	66.7%	0.861	Regazzo et al, 2016
miR-15b*, miR-23a, miR-133a, miR-150*, miR-197, miR-497 and miR-548b-5p	Downregulated	88%	97.87%	0.972	Yang et al, 2013
180 miRNA panel	NA	83%	79%	0.81	Roth et al, 2011
RNU6, miR-320, miR-574-3p	Upregulated and downregulated	87%	86%	0.926	Manterola et al, 2014
		70%	71%	0.722	Manterola et al, 2014
miR-15b-5p, miR-16-5p, miR-19a-3p, miR-19b-3p, miR-20a-5p, miR-106a-5p, miR-130a-3p, miR-181b-5p, miR-208a-3p	Upregulated	93.3%	94.5%	0.9722	Zhi et al, 2015
		94.0%	92.0%	0.9576	Zhi et al, 2015

### Sensitivity analysis

**Fig 4:**
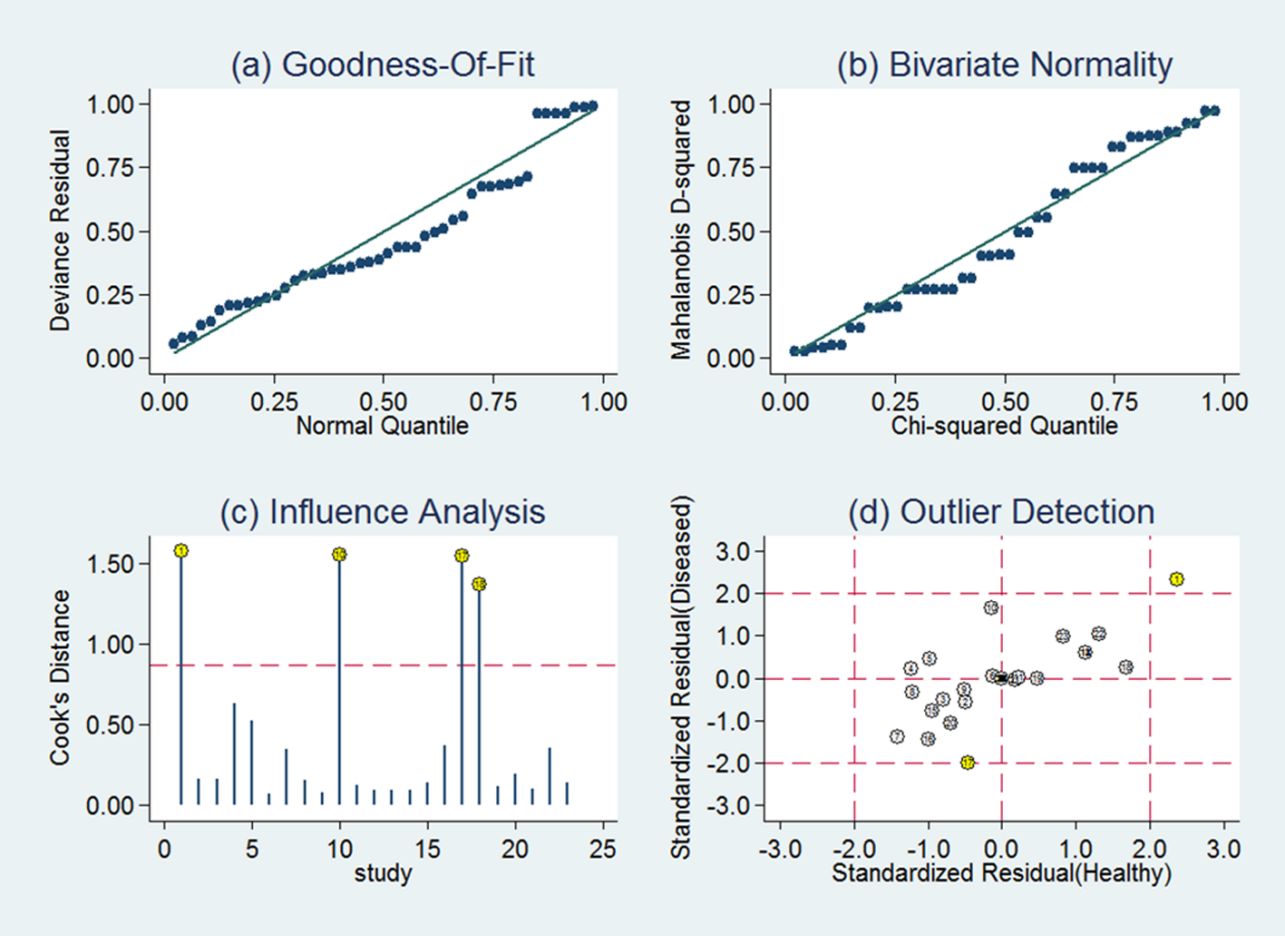
The diagram shows the (a) goodness-of-fit, (b) bivariate normality, (c) influence and (d) outlier detection analyses. Goodness-of-fit and bivariate normality showed this analysis fitted the model well. Influence analysis identified the most dominant studies in this meta-analysis. Outlier detection demonstrated one study (D’Urso et al and Xiao et al) is outside the standard residual square. Each number within circle represents the order of study identifier in Fig 3.

The goodness of fit included in our analysis shows a clear representation of the population. Q-Q plot (**Fig 4**) demonstrated the included studies were normally distributed despite a few outliers. Influence analysis showed that this meta-analysis was mainly contributed by the following studies D’Urso et al, Shao et al, Xiao et al and Yang et al in weight (**Fig 4**). Detection analysis identified D’Urso and Xiao **(Fig 4)**, indicating these two papers might be the source of heterogeneity in this meta-analysis. In addition, there is no potential publication bias detected by Deeks’ funnel plot asymmetry test (p = 0.53), as shown in the funnel plot **(S2 Fig)**.

## Discussion

In this meta-analysis study, we analyzed 16 publications relating to miRNAs as potential diagnostic markers of glioma. In our meta-analysis, the overall AUC was 0.93, suggesting that circulating miRNAs could be useful in detecting glioma. The overall specificity and sensitivity of circulating miRNAs was 0.87 and 0.86, respectively. This level of diagnostic accuracy is similar to that reported for proton magnetic resonance spectroscopy (specificity 0.87 and sensitivity 0.85) [37]. The pooled positive likelihood ratio of 6.39 indicated that the probability of glioma is 6-fold increased when the studied miRNAs was positive while the pooled negative likelihood ratio of 0.15 suggested that the probability of glioma decreased by 85% with negative miRNA testing. The diagnostic odds ratio of circulating miRNAs of 41.91 also showed an outstanding test performance. The validity of circulating miRNA for glioma diagnosis is the key point we need to address prior to its introduction into clinical practice. All studies included in this meta-analysis utilized qRT-PCR to detect circulating miRNA; this could be the best choice of technique for future application as it is highly sensitive for detecting low copies of miRNAs in serum samples, which is critical for routine test in clinical setting [38]. However, this meta-analysis identified a large heterogeneity of circulating miRNAs within all studies included (*I*^*2*^ = 87.89), which was likely due to data normalization.

Three miRNAs (miR-15b, miR-125b and miR-128) were identified by several studies [25, 26, 30, 33, 34] to be dysregulated in blood of glioma patients. In two of these studies [33, 34], miR-15b was found upregulated in glioma samples when compared to healthy controls. High levels of miR-15b in glioma tissue are strongly associated with poor prognosis [39] and biologically, miR-15b can induce cell cycle arrest, promoting cell proliferation and apoptosis by targeting cyclins [39, 40], suggesting its role in glioma development.

Serum miR-128 was also reported significantly dysregulated in glioma [23, 25, 26] and upregulated in patients post-surgery [23, 25]. In line with this finding, miR-128 has been reported to be downregulated in glioma tissues compared to normal brain tissue [41]; it is regarded as a critical regulator in glioma as it manipulates multiple biological phenotypes such as apoptosis, proliferation, angiogenesis, self-renewal, cell adhesion and chemotherapy resistance [41–45].

Serum miR-125b, was also commonly found at lower levels in high grade glioma patients compared to controls [27, 36]. However, in contrast, high levels of miR-125b expression are linked to malignancy in glioma cells [46], and it functions to regulate apoptosis via p53 and p38-MAPK and NF-κB pathways, common in glioma development. These opposite findings may be related to different roles or expression mechanisms for this miRNA in tissue vs serum and supports the need for careful selection of miRNA for glioma diagnosis prior to translating to clinical practice.

In the studies we retrieved here, some miRNAs were compared between pre-operative and post-operative patients. miRNAs including miR-205, miR-21, miR-128, miR-454, changed dramatically post-surgery [23, 24, 31], suggesting them as possible candidates for monitoring glioma progression. Our group recently published a study showing significant changes in circulating level of miR-21 and miR-10b in glioma patients undergoing treatment with an anti-angiogenic reagent, bevacizumab, indicating that these circulating miRNAs are potentially monitoring biomarkers for glioblastoma [47]. Another study showed that a panel of miR-21, miR-128 and miR-342 can be used as potential predictors of therapy response, as the levels of these circulating miRNAs were altered after chemo-radiation treatment [26]. However, post-operatively, serum miRNA levels are likely to reflect drug treatments and host factors as well as directly related to glioma progression, thus caution is required in interpreting this early evidence.

Several different endogenous controls were used in the studies analyzed, which possibly contributed to the heterogeneity of the data. There is no universal housekeeping control for miRNA and the process of choosing a suitable reference is still controversial [48]. It is known that RNU6 is widely used as an endogenous control in some cancer tissues or cellular samples, but it does not seem to work in bio-fluid samples [49][49][49][49], yet through this analysis, we have identified RNU6 as a reference control in several studies, which could likely cause misinterpretation in the dataset.

There are other confounding factors contributing to the heterogeneity within and between studies including the patients’ ethnic group, geographic location of the studies, and the statistical package used in data filtering and analysis. Another important factor affecting the future application of circulating miRNA in the clinic is the current lack of a standardized protocol for processing blood samples as well as RNA/miRNA isolation prior to qRT-PCR analysis, which is likely the main reason for the ‘high risk’ seen in applicability concerns of QUADAS-2 analysis.

A larger sample size may have improved the reliability of the conclusions in our meta-analysis, however, we are limited by the numbers of current published studies in this novel field of circulating miRNA in glioma. The sample size of 16 publications in our study is within the average range compared to meta-analyses of miRNA expression in various other cancers.

Based on our meta-analysis, circulating miRNAs can potentially be used as diagnostic tool for glioma with high sensitivity and specificity. The clinical role of serum miRNA analysis is likely to be in conjunction with MRI and histopathology at the time of diagnosis, as well as for monitoring for glioma recurrence post operatively. Towards this aim, we have recently performed a global miRNA profile analysis of serum from a large cohort of glioma patients in our local institution that has identified a set of miRNAs, some of which overlap with those in this meta-analysis. These biomarkers need to be validated in future clinical trials. Furthermore, a consensus needs to be established for a standardized sample processing and miRNA detection methods before introduction into clinical practice.

## Supporting information

S1 File Prisma Checklist. (DOC)

S1 Fig SROC curves describe the diagnostic performance of miRNAs in discriminating glioma from non-cancer subjects from plasma or serum sample. **A**) AUC is 0.96 (95%CI, 0.94–0.97) for plasma and **B**) AUC is 0.92 (95%CI, 0.89–0.94) for serum.
(TIF)

S2 Fig The Deek’s funnel plot of all included studies. This Deek’s funnel plot demonstrated that there is symmetry in all included studies, indicating low risk of publication bias in this meta-analysis, indicating (p = 0.53). Each number within circle represents the order of study identifier in Fig 3.
(TIF)
