# Supplementary figures and images for: A comprehensive meta-analysis of circulation miRNAs in glioma as potential diagnostic biomarker
Source: PLoS One. 2018 Feb 14;13(2):e0189452. doi: 10.1371/journal.pone.0189452 (PMC5812551; doi:10.1371/journal.pone.0189452)

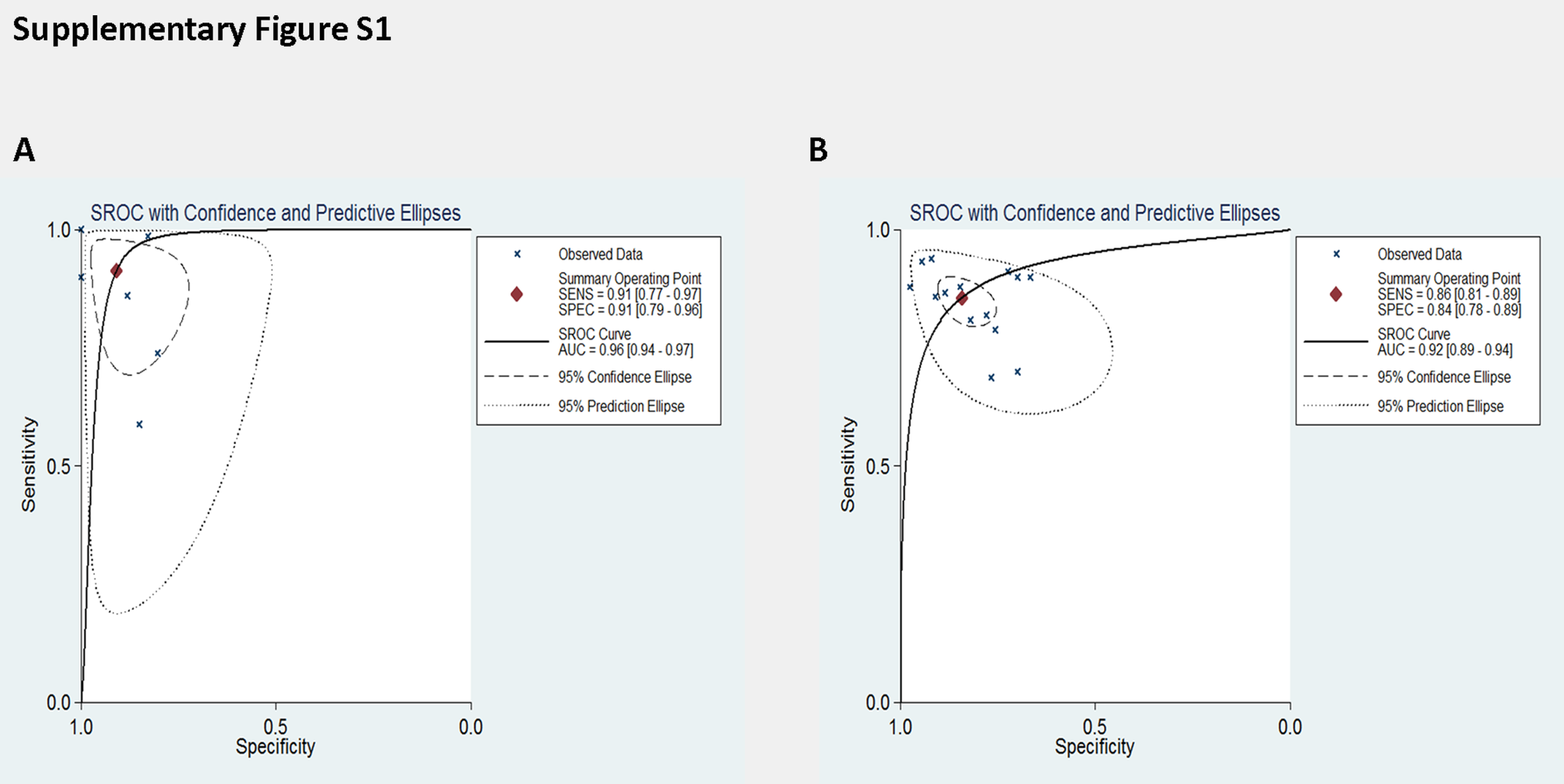

Supplement: S1 Fig — A) AUC is 0.96 (95%CI, 0.94–0.97) for plasma and B) AUC is 0.92 (95%CI, 0.89–0.94) for serum. (TIF) [file pone.0189452.s002.tif]

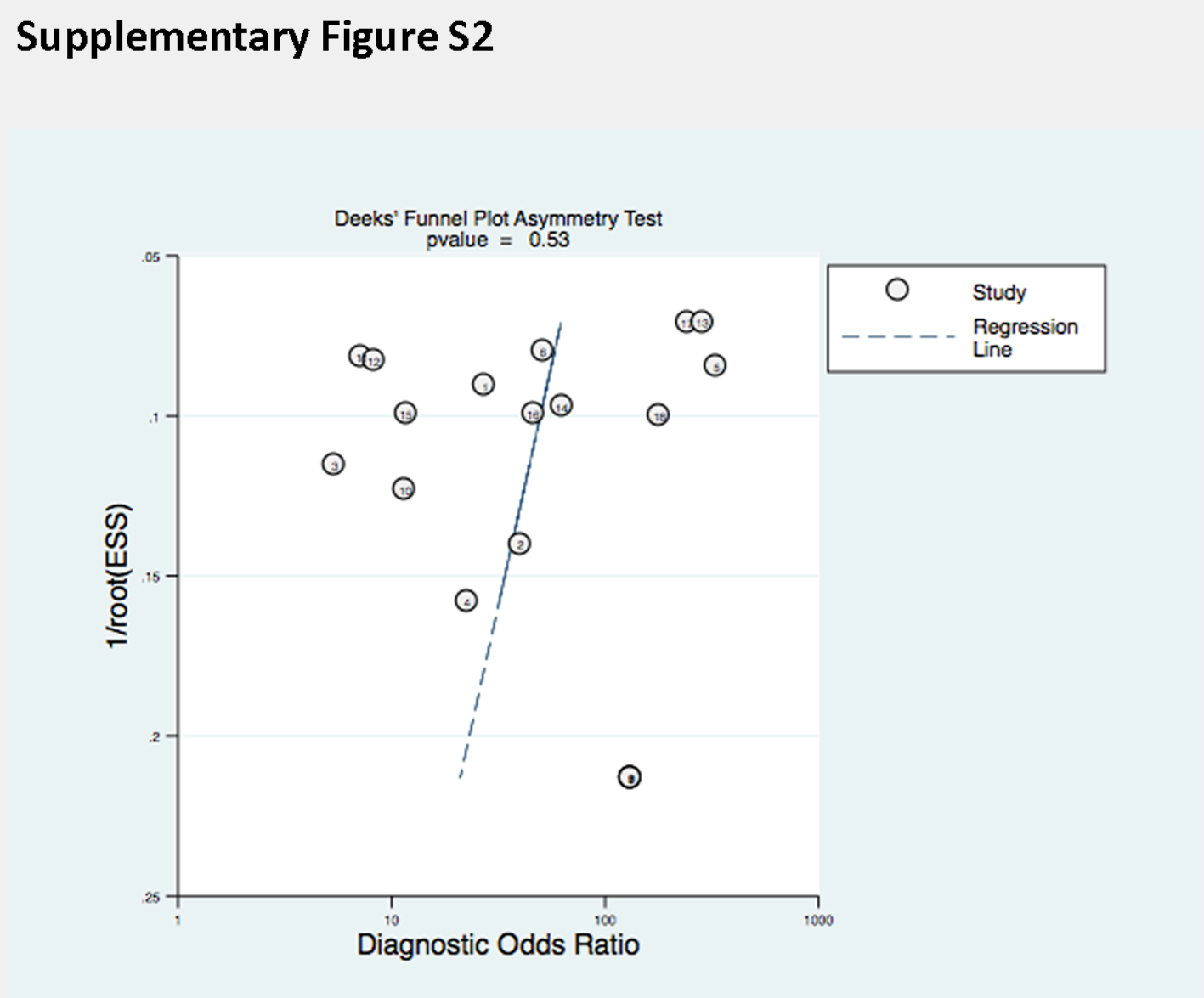

Supplement: S2 Fig — This Deek’s funnel plot demonstrated that there is symmetry in all included studies, indicating low risk of publication bias in this meta-analysis, indicating (p = 0.53). Each number within circle represents the order of study identifier in Fig 3. (TIF) [file pone.0189452.s003.tif]
